# Desynchronization and Wave Pattern Formation in MPI-Parallel and Hybrid Memory-Bound Programs

**DOI:** 10.1007/978-3-030-50743-5_20

**Published:** 2020-05-22

**Authors:** Ayesha Afzal, Georg Hager, Gerhard Wellein

**Affiliations:** 8 School of Computing, University of Utah, Salt Lake City, UT USA; 9 Cray, a Hewlett Packard Enterprise Company, Seattle, WA USA; 10 Helmholtz-Zentrum Dresden-Rossendorf, Dresden, Germany; 11 Extreme Computing Research Center, King Abdullah University of Science and Technology, Thuwal, Saudi Arabia; 12 Erlangen Regional Computing Center (RRZE), 91058 Erlangen, Germany; 13 Department of Computer Science, University of Erlangen-Nürnberg, 91058 Erlangen, Germany

## Abstract

Analytic, first-principles performance modeling of distributed-memory parallel codes is notoriously imprecise. Even for applications with extremely regular and homogeneous compute-communicate phases, simply adding communication time to computation time does often not yield a satisfactory prediction of parallel runtime due to deviations from the expected simple lockstep pattern caused by system noise, variations in communication time, and inherent load imbalance. In this paper, we highlight the specific cases of provoked and spontaneous desynchronization of memory-bound, bulk-synchronous pure MPI and hybrid MPI+OpenMP programs. Using simple microbenchmarks we observe that although desynchronization can introduce increased waiting time per process, it does not necessarily cause lower resource utilization but can lead to an increase in available bandwidth per core. In case of significant communication overhead, even natural noise can shove the system into a state of automatic overlap of communication and computation, improving the overall time to solution. The saturation point, i.e., the number of processes per memory domain required to achieve full memory bandwidth, is pivotal in the dynamics of this process and the emerging stable wave pattern. We also demonstrate how hybrid MPI-OpenMP programming can prevent desirable desynchronization by eliminating the bandwidth bottleneck among processes. A Chebyshev filter diagonalization application is used to demonstrate some of the observed effects in a realistic setting.

## Introduction

In principle, a parallel computer should be a deterministic system. Given some code and hardware specifications, it should be possible to predict the runtime of the program and measure it consistently in repeated experiments. Analytic, first-principles performance models such as Roofline [21] or ECM [10, 19] approximate this goal on the core and socket level. Although residual deviations and statistical variations remain, these models can yield valuable insights into the hardware bottlenecks of computation despite the simplifications that go into the model assumptions. One of these is the notion that all cores or hardware threads execute the same code on different data, which is often true for programs exploiting thread-level loop parallelism. With message passing, however, the dependencies among instruction streams (processes) are less tight, and communication overhead complicates the picture. Ideally, one would like to add communication models such as the Hockney model [7] or its refinements on top of Roofline or ECM, but this is too simplistic: System noise, variations in network bandwidth and latency, load imbalance, and strong one-off delays can cause global effects such as desynchronization and traveling idle waves [2, 15]. Using threaded MPI processes changes the phenomenology and dynamics of the system, because socket-level bottlenecks (i.e., memory bound vs. core bound) play a decisive role. It is therefore necessary to shed light on the dynamic processes that lead to desynchronization and global structure formation in pure MPI and threaded MPI programs.

An *idle wave* is a period of idleness caused by a strong delay in computation or communication on one process of an MPI program. It travels across the MPI processes with a speed that is governed by the particular communication characteristics (distance of communication, eager vs. rendezvous mode, etc.) and interacts with other idle waves, computational noise, and system noise in a nonlinear way. In this paper, we extend a previous study on the dynamics of idle waves with core-bound pure-MPI programs [2] towards the memory-bound case, i.e., codes with a low computational intensity. These exhibit saturation characteristics when running on multiple cores connected to a single memory interface (the *contention domain*^1^). The basic mechanisms are investigated using parallel microbenchmarks that are amenable to straightforward node-level performance modeling and can be easily altered to mimic different application characteristics.

We start by comparing the dynamics of traveling idle waves generated by injected one-off delays between core-bound and memory-bound MPI programs with negligible communication overhead and perfect load balance. More complex dynamics can be observed in the memory-bound case within the memory domain and when crossing domain boundaries (sockets, nodes). Even after the idle wave is gone, a distinctive “computational wave” pattern prevails that is governed by the topological properties of the MPI program (inter-process communication dependencies, boundary conditions) and the location of the memory bandwidth saturation point, i.e., the number of processes required for full memory bandwidth utilization. In case of significant communication overhead, a massive one-off delay is not required to provoke the wave pattern; the natural system noise or a single, small disturbance of regularity in computation or communication time is sufficient. Based on these observations, we study the impact of using threaded MPI processes. Multithreading has an influence on the bandwidth saturation point, and filling the contention domain with a multi-threaded MPI process effectively generates a bandwidth-scalable code. This answers the long-standing question why a nonreflective introduction of OpenMP threading into an MPI-only code can in some cases cause a slowdown even if OpenMP-specific overheads are negligible [5, 22]. Finally, we employ an application code implementing Chebyshev Filter Diagonalization (ChebFD) for a topological insulator problem to show the relevance of our findings in a real-world scenario.

The configuration parameter space of MPI and hybrid MPI-OpenMP parallel programs is huge. Here we restrict ourselves to simple, bidirectional point-to-point communication using eager or rendezvous protocols (depending on the message size).

This paper is organized as follows: Sect. 2 details our experimental environment and methodology. In Sect. 3 we study the propagation of an injected, one-off delays, contrasting memory-bound with core-bound scenarios. Computational wavefronts in memory-bound programs emerging from idle waves are covered in Sect. 4. Section 5 deals with the spontaneous formation of wavefronts and its consequences on program performance, and in Sect. 6 we showcase some of the observed effects using an application code. Section 7 covers related work and Sect. 8 concludes the paper and gives an outlook to future work.

*Contributions.* This work makes the following novel contributions:

- We show the characteristics of idle waves traveling through memory-bound MPI applications on multicore clusters, and how they differ from the core-bound case studied in prior work [2].
- We show that the forced emergence of computational wave patterns via desynchronization by one-off delays only occurs with memory-bound code.
- We show that the average available memory bandwidth per core in an established computational wave (desynchronized state) is larger than in the synchronous state while the core is executing application code. The wave settles in a state where the number of active processes per contention domain is near the memory bandwidth saturation point.
- We show how natural system noise leads to spontaneous desynchronization and computational wave formation if there is significant communication overhead.
- We show that desynchronization can lead to automatic overlap of communication and computation, reducing overall time to solution. Significant intra-node communication overhead can reduce this gain.
- We show that the introduction of threaded MPI processes can prevent the formation of computational waves and automatic communication overlap if one process is used per contention domain, effectively recovering the characteristics of a scalable pure MPI code.

## Experimental Environment and Methodology

### Cluster Test Bed and External Tools

In order to ensure that our observed phenomenology is not specific to a singular hardware or software setup, four different clusters were used to conduct various experiments:

- Emmy^2^, a QDR-InfiniBand cluster with dual-socket nodes comprising ten-core Intel Xeon “Ivy Bridge” CPUs ans Hyper-Threading (SMT) enabled,
- Meggie^3^, an Omni-Path cluster with dual-socket nodes comprising ten-core Intel Xeon “Broadwell” CPUs and Hyper-Threading (SMT) disabled,
- Hazel Hen^4^, a Cray XC40 with Aries interconnect and 12-core Intel Xeon “Haswell” CPUs,
- SuperMUC-NG^5^, an Omni-Path cluster with dual-socket nodes comprising 24-core Intel Xeon “Skylake SP” CPUs.

Details of the hardware and software environments on these systems can be found in Table 1.

**Table 1.** Key hardware and software specifications of systems

Systems	Emmy	Meggie	Hazel Hen (CRAY XC40)	SuperMUC-NG
Intel Xeon Processor	Ivy Bridge EP	Broadwell EP	Haswell EP	Skylake SP
Processor model	E5-2660 v2	E5-2630 v4	E5-2680 v3	Platinum 8174
Base clock speed	2.2 GHz	2.2 GHz	2.5 GHz	3.10 GHz(2.3 GHz used$*$)
Physical cores per	20	20	24	48
Dual socket node				
LLC size	25 MB	25 MB	30 MB	33 MB
Memory per node (type)	64 GB (DDR3)	64 GB (DDR4)	128 GB (DDR4)	96 GB (DDR4)
Theor. memory bandwidth	51.2 GB/s	68.3 GB/s	68.3 GB/s	128 GB/s
Node interconnect	QDR InfiniBand	Omni-Path	Cray Aries	Omni-Path
Interconnect topology	Fat-tree	Fat-tree	Dragonfly	Fat-tree
Raw bandwidth per	40 Gbits$^{-1}$	100 Gbits$^{-1}$	126 Gbits$^{-1}$	100 Gbits$^{-1}$
Link and direction				
Software				
Compiler	Intel v2019.4.243	Intel v2019.4.243	Cray v8.7.10	Intel v2019.4.243
Message passing library	Intel MPI v2019u4	Intel MPI v2019u4	Cray MPICH v7.7.6	Intel MPI v2019u4
Operating system	CentOS Linux v7.7.1908	CentOS Linux v7.7.1908	SESU Linux ENT. Server 12 SP3	SESU Linux ENT. Server 12 SP3
Tools				
ITAC	v2019u4	v2019u4	$\S$	v2019
LIKWID	5.0.0	5.0.0	$\S$	4.3.3

$*$ A power cap is applied on SuperMUC-NG, i.e., the CPUs run by default on a lower than maximum clock speed (2.3 GHz instead of 3.10 GHz).

$\S$  high-resolution Chrono clock for timing measurement.

We used Intel trace analyzer and collector (ITAC)^6^ for timeline visualization (except on Hazel Hen, where traces were recorded by explicit timing measurements), the  high-resolution Chrono clock for timing, and likwid-perfctr from the LIKWID tool suite^7^ for memory bandwidth measurements.

### Experimental Parameters and Methodology

We took a number of measures to create a reproducible experimental environment and minimize any noise from system sources. On the Emmy and Meggie systems, we ran all multi-node experiments on nodes connected to a single leaf switch. Core-thread affinity was enforced. The computational workload for the core-bound case was a number of back-to-back divide instructions (vdivpd), which have a low but constant throughput on Intel architectures if “simple” denominators are avoided. Except for the application case study, the memory-bound workload comprised simple kernels like STREAM triad. One-off idle periods were generated by massively extending one computational phase via doing extra work.

Most microbenchmark experiments were performed on two nodes only, since the basic phenomenology is visible even on this scale. Bidirectional point-to-point communication between MPI processes employed a standard MPI_Isend/MPI_IRecv/MPI_Waitall sequence. Before actual measurements were taken, at least two warm-up time steps with barrier synchronization were performed to allow the MPI and OpenMP runtimes to settle and eliminate first-call overhead. We only report statistical variation in measurements where the relative spread was larger than 5%. Unless otherwise stated, the clock speed of processors was fixed. On SuperMUC-NG the active power capping feature leads to an effective clock speed of 2.3 GHz, which was validated by the likwid-perfctr tool.

## Idle Wave Mechanisms for Memory-Bound Code

In [2], idle waves were shown to have *nonlinear* characteristics, i.e., colliding waves interact and partially cancel each other. Noise, i.e., short delays from different sources such as load imbalance, varying communication characteristics, or system noise, causes the decay of traveling idle waves. In this section, we compare the known dynamics of idle waves between core-bound code and memory-bound code. For brevity and to avoid confusion, we will call the two phenomena *core-bound* and *memory-bound idle wave*, respectively. We also restrict ourselves to the case of negligible communication overhead, i.e., a small communication-to-computation ratio.

### Idle Wave Propagation Speed

**Fig. 1.**
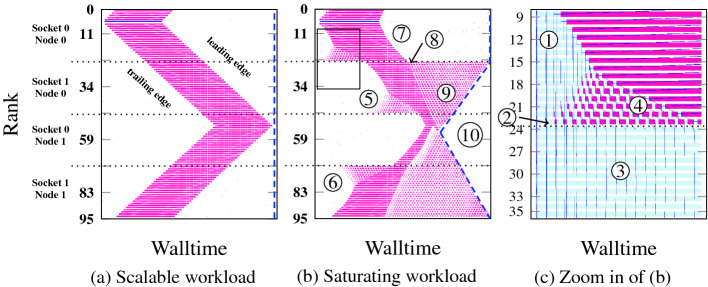
Timelines of idle waves through MPI code (one process per core) with different workload characteristics, negligible communication overhead, and bidirectional next-neighbor communication in a closed ring topology on SuperMUC-NG. The *y* axis is the MPI rank and the *x* axis is wall-clock time. Red indicates waiting time (within the MPI library) while white or light blue denote user code (50 iterations). The injected delay of about 25 execution phases is shown in dark blue. (a) Core-bound code with execution phase of 10 ms, (b) memory-bound STREAM triad code (overall data transfer volume of 4.8 GB, evenly distributed across all cores for a computation phase of 11.5 ms), (c) zoom-in of marked area in (b). (Color figure online)

Figure 1a shows a traveling idle wave on the SuperMUC-NG system with core-bound code. The leading and the trailing edges of the wave are parallel, and due to the communication characteristics (bidirectional next-neighbor, eager mode, closed ring) the waves emanating from the idle injection cancel each other after one half round trip. The memory-bound code in Fig. 1b shows a very different pattern: Since the available memory bandwidth per core declines after the saturation point, the length of any particular execution phase on any particular MPI process depends on how many other processes are executing user code at the same time on the same contention domain. If *b*(*N*) is the STREAM memory bandwidth with *N* processes, transferring a data volume of *V* bytes with a single process takes a time of

1 $$\begin{aligned} T_\mathrm {exec}=\frac{NV}{b(N)}. \end{aligned}$$

In the saturation phase, where $N>N_\mathrm {sc}$, $b(N)\sim \text {const.}$ and thus $T_\mathrm {exec}\sim NV$, i.e., as the front (back) of the idle wave progresses through the cores of a socket and more (fewer) cores participate in code execution, the time per iteration goes up (down). Hence, the forward and backward edges of the idle wave ripple through the system at *variable* propagation speeds.

The expression for the silent-system idle wave propagation speed from [2] still holds, but with modifications. Instead of the whole idle wave velocity, we can only draw conclusions for either of its two edges at a single moment in time since the execution time obeys the relation (1). The *local* velocity is

2 $$\begin{aligned} v_\mathrm {silent}(N) = \frac{\sigma \cdot d}{NV/b(N)+T_\mathrm {comm}}~\left[ \frac{\mathrm {ranks}}{\mathrm {s}}\right] , \end{aligned}$$

where *N* is the number of processes executing code. This means that $\nu _\mathrm {silent}$ can be different for processes on the same contention domain, which will be investigated further in the next section. $T_\mathrm {comm}$ is the communication time, *d* parameterizes the distance of communicating processes, and $\sigma \in \{1,2\}$ is a correction factor that depends on communication characteristics, e.g., communication patterns (uni- vs. bidirectional), flavors (multiple split-waits vs. one wait-for-all), and protocols (eager vs. rendezvous) [2].

Note that (2) even holds for hybrid MPI/OpenMP programs that communicate only outside parallel regions. In this case, *N* is the number of active multi-threaded MPI processes on a socket. If the process spans the full socket, $N=1$ and the propagation speed does not vary. This setting will be analyzed in Sect. 5.3.

### Idle Wave Decay

In [2] it was shown that noise, i.e., small statistical disturbances of the pure lock-step pattern, cause the decay of traveling idle waves, possibly to the point where a one-off injection does not even impact the time to solution of the program. In a noise-free system, a core-bound idle wave does not decay, but eventually interacts with itself or with the boundaries of an open process topology.

The propagation and decay mechanisms of memory-bound idle waves are much different since the propagation speed of the trailing and leading edges is strongly influenced by topological domain boundaries, specifically those between adjacent contention domains. Together with the contention effect, decay occurs even on a silent system. Figure 1(b, c) shows the basic phenomenology: As the idle wave progresses through the contention domain (from core 5 to 23 as shown in the upper section of Fig. 1b and in the upper half of Fig. 1c), the trailing edge is gradually getting steeper as fewer cores participate in the computation (cores 6–16  ) because more bandwidth becomes available per core. On the other hand, idle phases are emanating from the end of the domain (core 23  ) because the next contention domain (core 24 and up,  ) is still executing with all cores and is thus slower per core. These small idle waves propagate up and interact with the main idle wave on cores 17–23  , effectively causing its partial decay. The same occurs on the second contention domain at cores 39–47  and, in reverse direction due to the wrapping around of the wave, on the fourth domain on cores 72–80  .

Domain boundaries and the memory bottleneck are just as important for the leading edge dynamics. Within the domain where the one-off delay was injected (cores 6–23  ), the leading edge of the idle wave is not straight but shows a slowdown as time progresses. This is because the number of active cores on the contention domain increases as the wave propagates, and the available memory bandwidth per core goes down as soon as contention sets in. Eventually, the leading edge hits the boundary to the next contention domain. Right after this point  the first domain is free of any delay and the bulk-synchronous execution is restored there. The idle wave is now progressing entirely through the second domain. Since the first domain is in synchronous state and there is idleness on the second domain, more bandwidth is available per core on the latter, so the computation phases are shorter and there is waiting time (small red boxes in the timeline graph  ). The key observation here is that the second domain does not go back to a synchronized state; computation alternates with waiting times on every process, but enough cores are active concurrently to saturate the memory bandwidth. Hence, the overall throughput of the second domain is the same as on the first but the processes are out of sync. Finally, after the preset number of time steps has passed, the computation terminates. Processes that have collected less idle time because of the decay of the injected idle wave (on the second to fourth domain) finish early, as shown by the dashed blue line in Fig. 1b  . A distinctive wave-like pattern emerges across all contention domains but the one in which the idle wave was injected. We call this pattern a “computational wavefront.”

## Induced Computational Wavefronts

In this section, we will further analyze the generating mechanisms of computational wavefronts with memory-bound MPI code that emerge from singular one-off delays. We restrict ourselves to the case of negligible communication overhead. *Spontaneous* wavefront formation and significant communication overhead are linked and will be covered in Sect. 5.

**Fig. 2.**
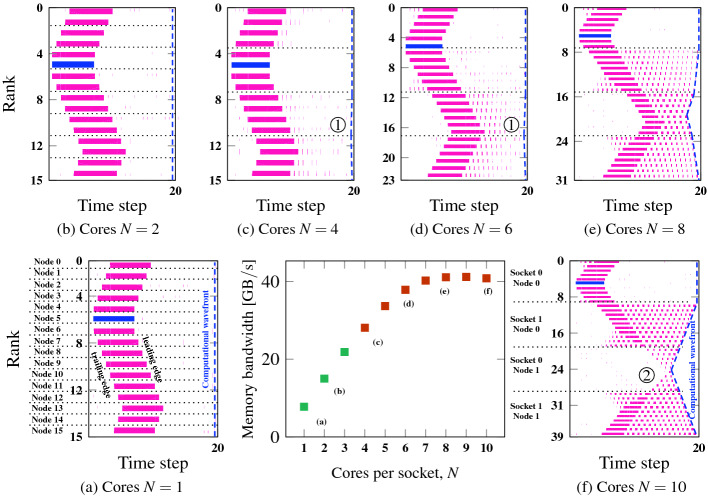
Idle wave-induced computational wavefront pattern formation with memory-bound (STREAM triad with nontemporal stores) workload on the Emmy system with varying number of MPI processes per contention domain (socket). Overall triad data volume was 9.6 GB, other parameters as in Fig. 1. Middle panel: memory bandwidth versus number of cores for the STREAM triad benchmark on one socket. (a)–(f) Idle wave propagation with 1,...,10 cores per contention domain over 20 time steps. Horizontal dashed lines denote socket boundaries except in (a), where one process per node was run on the first core of the first socket. Computational wavefronts are shown with blue dashed lines. (Color figure online)

### Wavefront Amplitude vs. Processes per Contention Domain

A computational wavefront is a stable structure that can be visualized by marking the wallclock time of a specific time step on each MPI process in a bulk-synchronous iterative application. In a fully synchronized state, the pattern is a straight line perpendicular to the time axis. Desynchronization causes wave-like patterns like the one shown in Fig. 1b. We have shown above that the memory-bound nature of the code is crucial for desynchronization, so we start with a series of experiments with progressively more severe memory bottlenecks. Figure 2 shows six timelines of memory-bound MPI programs on the Emmy system (parameters as in Fig. 1) after injecting a one-off delay. The difference among the six cases is the number of MPI processes per contention domain (socket). In the scalable regime (up to $N=3$ cores per socket) the idle wave causes no visible computational wave. As soon as the bandwidth bottleneck becomes relevant, i.e., when using more cores leads to less bandwidth available per core, (here at $N\gtrsim 4$), the damping effect on the idle wave sets in although it is weak at first (Fig. 2c,d). Our experiments show, however, that even in this regime a stable computational wave persists, albeit with a low amplitude  . At strong saturation ($N\gtrsim 7$) the fully developed wave is clearly visible. In all cases, the desynchronization prevails even after the idle wave has died out, and even on contention domains that were never traversed by it (cores 20–29 in Fig. 2f  ). Note also that the socket on which the idle wave was originally injected is still synchronized.

This shows that strong computational wave patterns require a strong memory bandwidth saturation. Note that wave patterns will also form without initial one-off idle injection, but this is a very slow process so we provoked it by “kicking” the system. This “kick” will not be required when there is significant communication overhead. See Sect. 5 for details.

**Fig. 3.**
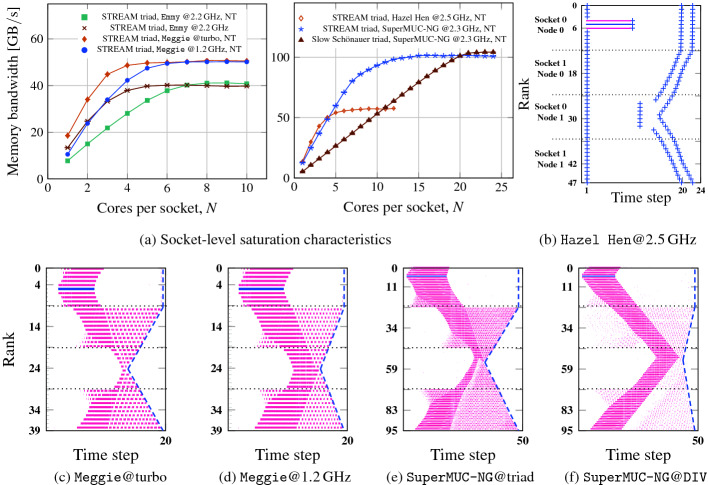
Saturation characteristics of benchmark platforms with different code and frequency settings and their influence on computational wave formation. (a) Bandwidth saturation of microbenchmarks on a contention domain (MPI strong scaling) on four systems: STREAM triad on Emmy with vs. without NT stores and on Meggie using Turbo Mode vs. lowest core frequency. On SuperMUC-NG, using STREAM triad and a “slow” Schönauer triad, and standard STREAM triad on Hazel Hen. (b)–(f) Timeline visualization of idle wave-induced computational wave emergence under different saturation conditions. On Hazel Hen, ITAC was not available so the trace was taken via explicit timing measurements.

**Fig. 4.**
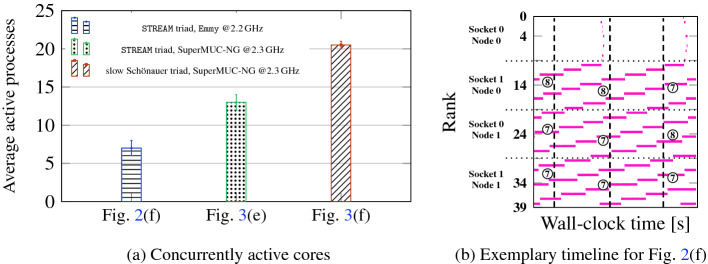
(a) Average number of MPI processes executing user code concurrently for the fully developed steady state computational waves (wavelength MPI_COMM_SIZE) in Figs. 2(f), 3(e), and 3(f). Minimum and maximum values among 60 samples along the timeline are indicated as whiskers. Data points were taken from the timeline data as shown in (b). Numbers in circles denote number of active processes at this point in time on this contention domain.

### Saturation Point and Wavefront Amplitude

There is still the question whether the saturation point, i.e., how many processes are needed to attain maximum memory bandwidth, plays any role. Our benchmark platforms exhibit different characteristics in this respect, as shown in Fig. 3a: The Broadwell CPUs on Meggie have the convenient property that the saturated memory bandwidth depends only weakly on the clock speed, so we set the core frequency to a constant 1.2 GHz or activated “Turbo Mode.” The latter led to clock frequency varying from 3.0 GHz (1 core) to 2.4 GHz (full socket) along the scaling curve. On SuperMUC-NG with its 24 cores per contention domain and fixed 2.3 GHz clock speed, we employed a modified variant of the Schönauer vector triad that has a higher computational cost (A(:)=B(:)+cos(C(:)/D(:))) in order to increase $N_\mathrm {sc}$ from about 14 to 20 cores. As a side effect, the saturation point becomes more sharply defined. On Emmy, using nontemporal (NT) stores for the STREAM triad the single-core bandwidth is about a factor of two lower than with standard stores, shifting the saturation point further out.

In Fig. 3b–f these variants are tested for their reaction to injected idle waves when using all cores on the contention domain. The data shows that the more data hungry the serial code (i.e., the earlier the saturation point), the stronger the damping. This was expected from the analysis in Fig. 1. In addition, an early saturation point causes a large amplitude of the generated computational wavefront (compare Fig. 3c and d, and Fig. 3e and f). Thus, the saturation point impacts the amplitude of the computational wavefront. Since the wavefront is defined by a constant time step ID across processes, a large wave amplitude indicates a larger inter-process skew, i.e., stronger desynchronization, which causes longer waiting times within MPI calls despite negligible communication volume. Since the computational wave survives even long after the idle wave has died out, it is impossible for these waiting times to cause reduced memory bandwidth utilization (else the still-synchronized contention domain would eventually catch up). It thus seems that there are is always a sufficient number of computing processes within the computational wave to still reach bandwidth saturation. Figure 4a shows the average number of computing processes within the fully developed wave for the three cases in Figs. 2(f), 3(e), and 3(f). Comparing with Fig. 3a it is evident that this number is very close to the bandwidth saturation point (at 7, 13, and 20 cores, respectively). Hence, the computational wave settles at an amplitude that allows for just enough active processes to saturate the memory bandwidth, but not more. The inevitable waiting times caused by desynchronization are perfectly overlapped with user code execution.

**Fig. 5.**
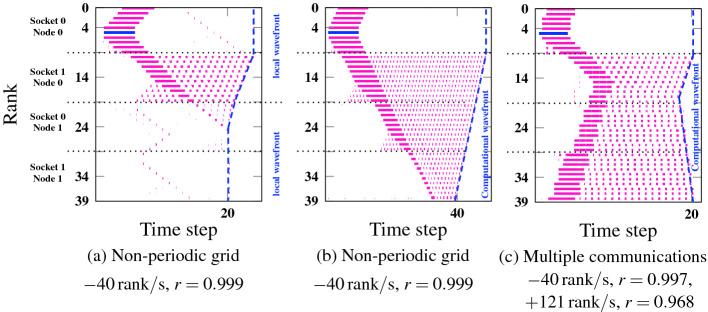
Shape and slope of memory-bound computational wavefront with different communication topologies and patterns on the Emmy system. The measured slope(s) of the computational wave(s) in ranks per second is/are indicated together with correlation coefficients of linear fits. Code properties are the same as in Fig. 2. The *x* axis shows walltime but the time step at which the computation was terminated is indicated. (a) Open boundary conditions, next-neighbor communication, short one-off idle injection, (b) open boundary conditions, next-neighbor communication, long one-off idle injection, (c) periodic boundary conditions, next-neighbor communication along rising ranks, next- and next-to-next neighbor communication along falling ranks, short one-off idle injection.

### Influence of Communication Patterns and Injection Length

In Fig. 5 we investigate how the shape and slope of an induced computational wave depends on the communication pattern (distance of point-to-point communication) and topology (open vs. periodic boundary conditions). In Fig. 5a we injected a short idle period into a code with open boundary conditions and next-neighbor bidirectional communication. The corresponding idle wave in negative rank direction dies at rank 0, as expected [2]. The idle wave in the positive rank direction hardly travels beyond the next contention domain (node 0, socket 1) before dying out, but a computational wave prevails on that domain in the form of a single ramp with a slope of $-40\,\text {rank/s}$. Doubling the duration of the injection (Fig. 5) leads to a longer idle wave that extends across three sockets in positive rank direction, and so does the generated computational wave. Its slope, however, is the same as in the previous case. The strength of the initial idle wave thus has no influence on the local slope of the computational wave.

The experiment in Fig. 5c shows the influence of communication patterns. Each MPI process communicates with its next neighbor in positive rank direction and with its next- and next-to-next neighbors in negative rank direction; moreover, the topology was changed to periodic boundary conditions. The idle wave can now roll over the system boundary and eventually annihilates itself. Its leading edges are governed by the known mechanisms investigated in [2]: The idle wave in negative rank direction is three times faster than the one in positive rank direction. The resulting computational wave is continuous (because of the boundary condition) and shows two distinct slopes, which are different from the slopes of the idle wave but have the same 3:1 ratio. Hence, the slopes involved in the computational wave are influenced by the same communication parameters that govern the slopes of the idle wave, but the absolute slopes are different, which translates into different wave amplitudes. As shown in the previous sections, they depend on the saturation characteristics of the memory-bound code.

## Spontaneous Computational Wavefronts

With negligible communication overhead, the desynchronization phenomena described above can be observed when provoked by a rather strong one-off delay injection. They only occur spontaneously, i.e., via the normal system noise, over very long time scales. Moreover, although the available memory bandwidth per process is larger in the desynchronized state, the runtime of the whole program, i.e., the wall-clock time required for the slowest process to reach the last time step, cannot be reduced in this scenario since no significant overhead is overlapped with code execution.

In this section we show how computational wavefronts and desynchronization can occur *spontaneously* via natural system noise if there is significant communication overhead, which paves the way towards automatic communication-computation overlap.

### Pure MPI

**Fig. 6.**
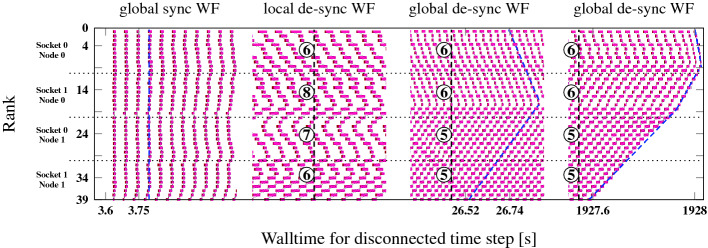
$50\,000$ iterations run of an MPI-parallel STREAM triad code (non-periodic grid, bidirectional next-neighbor communication, 4.8 GB overall data volume) on the Emmy system (normal stores, saturation at 5–6 cores). The four phases show different cutouts of the complete timeline near the indicated walltimes. Synchronized state (phase 1): 30 + 10 ms average compute + communication intervals. Fully developed wavefront (phase 3, 4): 20 + 17.5 ms average compute + communication. Numbers of concurrent working processes per domain are indicated in circles.

In Fig. 6 we show four phases of a timeline of a memory-bound STREAM triad code on four sockets of Emmy and an initial communication overhead of $\approx$25%. One MPI process was run per core with bidirectional next-neighbor communication, open boundary conditions, and a message size of 5 MB. The synchronized state from the beginning soon dissolves. After 100 time steps (second phase), local wavefronts have emerged, but no global state is reached yet. Within 500 time steps (third phase), a global wave has formed, and it persists till the end of the program (50 000 time steps). Interestingly, although the wavelength and amplitude of the computational wave are rather constant, the pattern can shift across the MPI ranks over time: After 26 s of walltime the slowest process is on socket 1, while after 2000 s it is on socket 0. The cause for such shifts are small perturbations (natural noise), whose close investigation is left for future work.

The overall MPI time per process goes up when entering the wave state as expected because waiting time is added on top of actual communication time. However, since communication can be overlapped with execution, performance increases. In our particular case, the total average (computation plus communication/waiting) time per iteration goes down from $30\,\text {ms}+10\,\text {ms}=40\,\text {ms}$ to $20\,\text {ms}+17.5\,\text {ms}=37.5\,\text {ms}$, i.e., by about 6%.

### Latency- vs Bandwidth-Dominated Overhead

There are two potential benefits from desynchronization: Better memory bandwidth utilization by the application code and better network interface utilization (not discussed here). These advantages are partially offset by the memory bandwidth drawn by MPI communication of large messages. For example, in the experiment in Fig. 6, each message had a size of 5 MB. In particular the intra-node point-to-point communication can aggregate to a significant data volume (at least 20 MB per process and time step in this case, and probably more depending on the implementation of intra-node MPI), reducing the bandwidth available to the application code. This is why the theoretical speedup of 25% could not be obtained.

### Threaded MPI Processes

All phenomenology discussed so far can also be observed with hybrid MPI+OpenMP codes that communicate only outside OpenMP-parallel regions. However, spanning an MPI process across several cores on a contention domain is equivalent to reducing the number of cores, which makes for weaker saturation characteristics as discussed in Sects. 4.1 and 4.2. If the number of threads per process is large enough to show linear bandwidth scaling across processes, spontaneous wave formation and automatic overlap will not occur.

**Fig. 7.**
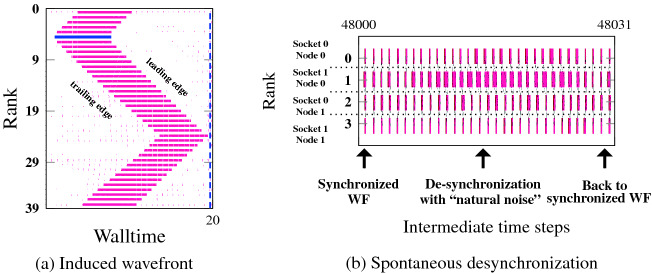
MPI+OpenMP hybrid execution of parallel STREAM triad on Emmy with bidirectional next-neighbor communication, periodic boundary conditions, and the same overall data volume as in Fig. 6 but with 10 threads per process and one process per contention domain. (a) 40 processes on 20 nodes with negligible communication overhead and an idle injection on process 5 for first 20 iterations, (b) four processes on two nodes with 5 MB MPI message size for intermediate 31 iterations over a complete run of 50 000 timesteps.

Figure 7a shows an injected idle wave on Emmy with 40 MPI processes by ten threads each, running the STREAM triad with one process per contention domain, bidirectional next-neighbor communication (negligible overhead), and periodic boundary conditions. Since there is no bandwidth contention among processes, the situation is very similar to Fig. 1 and Fig. 2a: The idle wave is hardly damped and eventually cancels itself, with no discernible desynchronization prevailing and no computational wave following up. The memory-bound nature of the code is of no significance.

The property of scalable code to automatically eliminate idle waves by the interaction of the trailing edge with system noise (which was thoroughly studied in [2]) leads to the important and general conclusion that spontaneous desynchronization does not occur in this case. Figure 7b shows a timeline of four MPI processes with ten threads each, running on four contention domains of Emmy. System noise causes a delay with subsequent desynchronization, which is quickly dissolved and the system returns to the synchronized state. One can argue that there is more to hybrid MPI+OpenMP programming than optimizing communication overhead; “full hybrid” codes, in which one MPI process spans a full contention domain (or more), do not profit from desynchronization and automatic overlap since they enforce a lock-step across threads.

We have to add that we have deliberately chosen a simplified scenario where the number and size of point-to-point messages sent between processes does not depend on the number of threads per process. In real-world codes, many effects complicate matters, especially when comparing pure MPI with MPI+OpenMP code for the same problem since the number of messages and (probably) the communication volume changes [18]. A thorough study of this problem area is left for future work.

## Chebychev Filter Diagonalization

Chebyshev filter diagonalization (ChebFD) [17] is a popular technique for calculating inner or extremal eigenvalues of large sparse matrices. It is based on subspace projection via polynomial filters constructed from Chebyshev polynomials. ChebFD is applied in many problems in quantum physics and chemistry, such as the study of topological materials (e.g., graphene) or electronic structure calculations based on density functional theory. Although the basic algorithm is just a sequence of simple vector operations and sparse matrix-vector multiplications (SpMV), it is amenable to loop fusion and blocking optimizations [12].
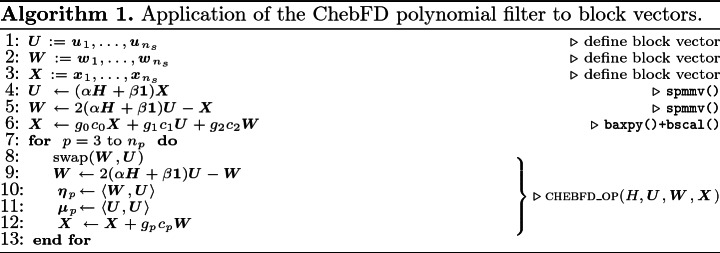


We use the scalable ChebFD implementation, specifically the application of the polynomial filter to a block of vectors. The compute kernels and implementation alternatives are available with the open-source GHOST^8^ library for download. This is the dominant part of the full ChebFD algorithm, which still requires an orthogonalization procedure that is omitted here without loss of generality. The code supports MPI+OpenMP parallelism.

Algorithm 1 shows the basic algorithm. *H* is the Hamiltonian matrix describing the physical system, while *U*, *W*, and *X* are blocks of $n_s$ vectors, with $n_s$ being the dimension of the search space. The loop from line 7 to 13 iterates up to the polynomial degree $n_p$, which determines how selective the polynomial filter will be. The goal of the algorithm is the computation of the polynomial filter coefficients $\{\eta _p\}$ and $\{\mu _p\}$, which requires global scalar products (lines 10 and 11). However, since these coefficients are not needed until after the end of the calculation, the global reduction can be postponed and leads to an algorithm without synchronization points or global operations. The body of the *p* loop can then be fused completely into a single kernel CHEBFD_OP for better cache reuse. Our implementation uses a blocking optimization that processes blocks of $n_b$ vectors at a time for improved cache efficiency. Details can be found in [12].

**Fig. 8.**
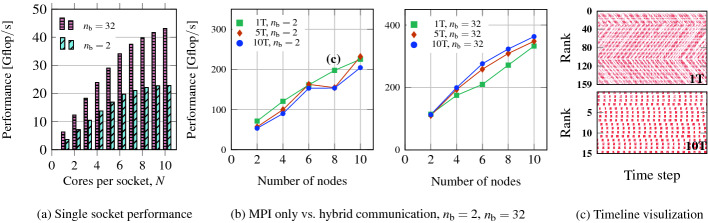
ChebFD application for the topological insulator matrix Topi-128-64-64 (static OpenMP scheduling, AVX vectorized and aligned execution, $n_\mathrm {iter}=5$) running on (single leaf switch connected) homogeneous Emmy nodes. (a) Performance scaling with OpenMP on a contention domain for $n_b=2$ and $n_b=32$, (b) scaling up to 10 nodes for $n_b=2$ and $n_b=32$, and different numbers of threads per process, (c) timeline for a specific number of iterations of pure MPI vs. full hybrid execution for $n_b=2$ and 8 Emmy nodes.

Our specific application case is a topological insulator of size $128\times 64\times 64$ with periodic boundary conditions. This leads to a Hamiltonian of dimension $2^{21}$ and $2.71\times 10^6$ nonzeros. The full working set is about 6.7 GB (double precision matrix, 4-byte indices, plus all block vectors) when using $n_s=128$ search vectors and a polynomial filter degree $n_p=500$, which are realistic values. The optimistic code balance assuming perfect cache reuse on the block vectors is [12]

3 $$\begin{aligned} B_c = \frac{260/n_b+80}{146}\frac{\mathrm{byte}}{\text {flop}}, \end{aligned}$$

which is well beyond the machine balance of all current CPUs even for large $n_b$, rendering the code memory bound according to a naive Roofline model. In reality, the $n_b=32$ case is already close to core bound since intra-cache data transfers begin to limit the performance of the code on some platforms, such as Emmy [13]: Fig. 8a shows performance vs. cores per socket for $n_b=2$ and $n_b=32$, and indeed the latter cannot fully saturate the bandwidth and achieves only 41 Gflop/s out of the bandwidth-bound Roofline limit of 66 Gflop/s. Figure 8b shows strong scaling from 2–10 nodes for both cases with 2 (10 threads each) to 20 (single-threaded) MPI processes on each Emmy node. At $n_b=2$, fewer threads have a clear advantage while the situation is reversed at $n_b=32$. The more saturating code ($n_b=2$) has ample opportunity for desynchronization without threading (which is shown in the timeline comparison in Fig. 8c). In Fig. 8c, the upper panel shows MPI only while the lower panel shows hybrid with 10 threads (1 process) per socket, both on eight Emmy nodes. The more scalable code ($n_b=32$) shows no spontaneous desynchronization without threading, and the fully hybrid code can benefit from the reduced number of MPI messages.

## Related Work

There is very little research on idle wave propagation and pattern formation in parallel code, especially in the context of memory-bound programs. Hence, none of the existing prior work addressed spontaneous pattern formation and desynchronization. Markidis et al. [15] used a simulator to study idle waves in MPI programs and their propagation for the first time. They did not consider the socket-level character of the code, though, and assumed a linear wave equation to govern the propagation of the waves. Afzal et al. [1, 2] have investigated the dynamics of idle waves in pure MPI programs with core-bound code. Our work builds on theirs and significantly extends it towards memory-bound code and spontaneous pattern formation. Gamell et al. [6] noted the emergence of idle waves in the context of failure recovery and failure masking of stencil codes, but the speed of propagation, the memory-bound characteristics of the application, and the corresponding damping mechanisms were not studied. Böhme et al. [4] presented a tool-based approach to attribute propagating wait states in MPI programs to their original sources, helping to identify and correct the root issues. Global properties of such waves like damping and velocity, or the interaction with memory-bound code, were ignored, however.

There is a vast body of research that targets the characterization of noise as well as its mitigation via explicit techniques, such as dynamic load balancing, MPI process placement, synchronization of OS influence, and lightweight OS kernels [3, 14, 16, 20]. In contrast, the present paper investigates the *favorable* consequences of noise as an enabling factor for desynchronization and – in case of memory-bound code – automatic partial overlap of communication and computation.

## Conclusion and Outlook

We have shown how the memory-bound nature of load-balanced MPI programs without explicit synchronization or global operations and homogeneous communication characteristics is directly linked to the damping of idle waves and to desynchronization effects. The key concept is the *computational wave*, a stable pattern marked by different processes reaching a given step within an application run at different times. Such patterns can be provoked by injected one-off delays or emerge spontaneously; rapid, spontaneous pattern formation caused by natural system noise is only possible with significant communication overhead. In a desynchronized state, the time spent in MPI routines is larger but the available memory bandwidth per process is higher. There is evidence that a computational wave settles in a state where the number of processes concurrently running user code within a contention domain is very close to the bandwidth saturation point. Desynchronization also enables automatic hiding of communication overhead, which can in some cases improve the performance of a program. This overlap may not be perfect due to the MPI communication requiring part of the memory bandwidth.

From the viewpoint of memory bandwidth, using a single, multi-threaded MPI process per contention domain effectively recovers a scalable code. In this case, automatic overlap does not occur and (induced or spontaneous) delays die out automatically. Multi-threaded MPI processes pay off mainly at larger core counts, where applications become more communication-intensive in strong scaling scenarios [5]. One main benefit from using threaded processes is a reduction in the number of messages. Thus, fewer threads per process can improve performance if process desynchronization can be leveraged for communication overlap without too much impact on the communication efficiency.

We consider studying microbenchmarks and simple applications as a necessary prerequisite to understand basic mechanisms. While our results were first obtained using simple microbenchmark codes on four different cluster systems, we have demonstrated the emergence of computational waves and the detrimental effect of full hybrid mode using a Chebyshev Filter application from quantum physics. Our coverage of the topic is certainly limited to the “barrier-free bulk-synchonous” pattern, i.e., regular communication-computation phases without explicit or implicit synchronization.

*Future Work.* Although we could uncover some of the mechanisms behind the computational wave formation in a qualitative way, a detailed quantitative understanding of these effects is still out of reach. For example, the length of computation and communication phases influences the idle wave velocity according to Eq. (2). A higher idle wave velocity will cause a smaller computational wave amplitude. Currently this is just an observation and we lack a quantitative model. Additionally, there is no actual mathematical *proof* of stability for computational waves, or a proof of instability for the bulk-synchronous state. We have also just scratched the surface of how threaded MPI processes, natural system noise, and network contention change the underlying mechanisms. For example, even with core-bound code there may be a strong bottleneck on the network interface if parallel program is strongly communication bound, and desynchronization does occur in this case as well. It will be helpful to have a controlled, noise-free experimental environment in which all relevant aspects, from code characteristics to communication parameters and contention effects, can be influenced at will. Well-known networking simulation tools [11], e.g., SST^9^ or CODES^10^, cannot accurately take resource sharing beyond network aspects into account since that would require a separate performance model on the node, such as the ECM model [9]. We are currently working on a high-performance simulation tool that goes far beyond existing simulators such as LogGOPSim [8].

## Footnotes

1 This is usually identical to a ccNUMA domain and often, but not always, a full CPU socket.

2 https://anleitungen.rrze.fau.de/hpc/emmy-cluster.

3 https://anleitungen.rrze.fau.de/hpc/meggie-cluster.

4 https://hlrs.de/systems/cray-xc40-hazel-hen.

5 https://doku.lrz.de/display/PUBLIC/SuperMUC-NG.

6 https://software.intel.com/en-us/trace-analyzer.

7 http://tiny.cc/LIKWID.

8 https://bitbucket.org/essex/ghost.

9 http://sst-simulator.org/.

10 https://press3.mcs.anl.gov/codes/.

## References

[1] Afzal, A., Hager, G., Wellein, G.: Delay flow mechanisms on clusters. In: Poster at EuroMPI: 10–13 September 2019, Zurich, Switzerland (2019). https://hpc.fau.de/files/2019/09/EuroMPI2019_AHW-Poster.pdf

[2] Afzal, A., Hager, G., Wellein, G.: Propagation and decay of injected one-off delays on clusters: a case study. In: 2019 IEEE International Conference on Cluster Computing, CLUSTER 2019, Albuquerque, NM, USA, 23–26 September 2019, pp. 1–10 (2019). 10.1109/CLUSTER.2019.8890995

[3] Bhatele, A., Mohror, K., Langer, S.H., Isaacs, K.E.: There goes the neighborhood: performance degradation due to nearby jobs. In: Proceedings of the International Conference on High Performance Computing, Networking, Storage and Analysis SC 2013, pp. 1–12 (2013). 10.1145/2503210.2503247

[4] Böhme D (2016). Identifying the root causes of wait states in large-scale parallel applications. ACM Trans. Parallel Comput..

[5] Chorley MJ, Walker DW (2010). Performance analysis of a hybrid MPI/OpenMP application on multi-core clusters. J. Comput. Sci..

[6] Gamell, M., et al.: Local recovery and failure masking for stencil-based applications at extreme scales. In: Proceedings of the International Conference for High Performance Computing, Networking, Storage and Analysis SC 2015, pp. 1–12, November 2015. 10.1145/2807591.2807672

[7] Hockney RW (1994). The communication challenge for MPP: Intel Paragon and Meiko CS-2. Parallel Comput..

[8] Hoefler, T., Schneider, T., Lumsdaine, A.: LogGOPSim - simulating large-scale applications in the LogGOPS model. In: Proceedings of the 19th ACM International Symposium on High Performance Distributed Computing, pp. 597–604. ACM, Chicago, June 2010. 10.1145/1851476.1851564. ISBN: 978-1-60558-942-8

[9] Hofmann J, Hager G, Fey D, Yokota R, Weiland M, Keyes D, Trinitis C (2018). On the accuracy and usefulness of analytic energy models for contemporary multicore processors. High Performance Computing.

[10] Hofmann, J., et al.: Bridging the architecture gap: abstracting performance-relevant properties of modern server processors. arXiv (2019, Submitted). arXiv:1907.00048 [cs.DC]

[11] Kenny JP, Sargsyan K, Knight S, Michelogiannakis G, Wilke JJ, Yokota R, Weiland M, Keyes D, Trinitis C (2018). The pitfalls of provisioning exascale networks: a trace replay analysis for understanding communication performance. High Performance Computing.

[12] Kreutzer M, Yokota R, Weiland M, Keyes D, Trinitis C (2018). Chebyshev filter diagonalization on modern manycore processors and GPGPUs. High Performance Computing.

[13] Kreutzer, M., et al.: Performance engineering of the Kernel Polynomial Method on large-scale CPU-GPU systems. In: 2015 IEEE International Parallel and Distributed Processing Symposium, pp. 417–426, May 2015. 10.1109/IPDPS.2015.76

[14] León, E.A., Karlin, I., Moody, A.T.: System noise revisited: enabling application scalability and reproducibility with SMT. In: 2016 IEEE International Parallel and Distributed Processing Symposium (IPDPS), pp. 596–607 (2016). 10.1109/IPDPS.2016.48

[15] Markidis S (2015). Idle waves in high-performance computing. Phys. Rev. E.

[16] Petrini, F., Kerbyson, D.J., Pakin, S.: The case of the missing supercomputer performance: achieving optimal performance on the 8,192 processors of ASCI Q. In: 2003 ACM/IEEE Conference on Supercomputing, pp. 55–55. IEEE (2003). 10.1145/1048935.1050204

[17] Pieper A (2016). High-performance implementation of Chebyshev filter diagonalization for interior eigenvalue computations. J. Comput. Phys..

[18] Rabenseifner, R., Hager, G., Jost, G.: Hybrid MPI/OpenMP parallel programming on clusters of multi-core SMP nodes. In: 2009 17th Euromicro International Conference on Parallel, Distributed and Network-based Processing, Los Alamitos, CA, USA, pp. 427–436. IEEE Computer Society, Feburary 2009. 10.1109/PDP.2009.43

[19] Stengel, H., Treibig, J., Hager, G., Wellein, G.: Quantifying performance bottlenecks of stencil computations using the execution-cache-memory model. In: Proceedings of the 29th ACM International Conference on Supercomputing, ICS 2015, Newport Beach, CA. ACM (2015). 10.1145/2751205.2751240

[20] Weisbach H, Gerofi B, Kocoloski B, Härtig H, Ishikawa Y, Yokota R, Weiland M, Keyes D, Trinitis C (2018). Hardware performance variation: a comparative study using lightweight kernels. High Performance Computing.

[21] Williams S, Waterman A, Patterson D (2009). Roofline: an insightful visual performance model for multicore architectures. Commun. ACM.

[22] Wu, X., Taylor, V.: Using processor partitioning to evaluate the performance of MPI, OpenMP and hybrid parallel applications on dual-and quad-core Cray XT4 systems. In: The 51st Cray User Group Conference (CUG2009), pp. 4–7 (2009). http://faculty.cse.tamu.edu/wuxf/papers/cug09.pdf

